# Structural determinants of protease-activated receptor 1 cleavage by activated protein C

**DOI:** 10.1016/j.jtha.2025.05.034

**Published:** 2025-06-09

**Authors:** Bosko M. Stojanovski, Enrico Di Cera

**Affiliations:** Edward A. Doisy Department of Biochemistry and Molecular Biology, Saint Louis University School of Medicine, St. Louis, Missouri, USA

**Keywords:** anticoagulant, protease-activated receptor, protein C, thrombin, serine protease

## Abstract

**Background:**

Activated protein C (APC) performs cytoprotective functions mediated by cleavage of the protease-activated receptor 1 (PAR1) in the presence of the endothelial protein C receptor and signaling through β-arrestin-2. APC cleaves PAR1 at R41 and R46, but the specificity of the reaction is low. In contrast, thrombin cleaves PAR1 at R41 only in a reaction that is independent of endothelial protein C receptor, producing a proinflammatory response mediated by signaling through G-protein intermediates and features high specificity. The molecular basis of this difference between APC and thrombin remains unknown.

**Objectives:**

To identify the structural determinants of APC that influence PAR1 specificity.

**Methods:**

Using available structural information, we engineered thrombin determinants of PAR1 recognition into APC. Specifically, we replaced T99 with Leu and swapped the entire 37- and 60-loops of APC with those of thrombin (eg, APC_60/T99L_ and APC_37/60/T99L_).

**Results:**

The engineered APC variants featured up to 80-fold enhanced specificity toward PAR1 mediated by increased cleavage at R41 and decreased cleavage at R46. Notably, the variants APC_60/T99L_ and APC_37/60/T99L_ also showed significantly reduced activity toward factor Va.

**Conclusion:**

The 37, 60, and 99 segments of APC determine the cytoprotective and anticoagulant properties of the enzyme.

## 1 | INTRODUCTION

In addition to its procoagulant and prothrombotic roles mediated by cleavage of fibrinogen and the protease-activated receptor 1 (PAR1), thrombin functions as a potent inhibitor of coagulation by activating the zymogen protein C (PC) into the enzyme activated PC (APC), which itself is endowed with diverse physiological roles [1]. As a natural anticoagulant, APC inactivates factor (F)Va and FVIIIa. As an anti-inflammatory and cytoprotective agent, APC signals through PAR1 to reduce cellular damage following sepsis and ischemia/reperfusion [2,3]. The cytoprotective functions of APC [2,3] depend on cleavage of PAR1 in the presence of the endothelial PC receptor (EPCR) [2] and signaling through β-arrestin-2 [4,5]. PAR1 is also activated by thrombin independently of EPCR and triggers a signaling pathway mediated by G-protein intermediates, resulting in a proinflammatory response and disruption of the endothelial barrier integrity [2,4,5]. How cleavage of the same receptor, PAR1, leads to distinct signaling pathways [6] remains unresolved at the molecular level. It has been proposed that PAR1 is cleaved at different sites by thrombin and APC, with the former cleaving exclusively at R41 and the latter cleaving at both R41 and R46 [7–9]. Cleavage at R46 is responsible for the beneficial cytoprotective function of APC [7,8].

**FIGURE 1:**
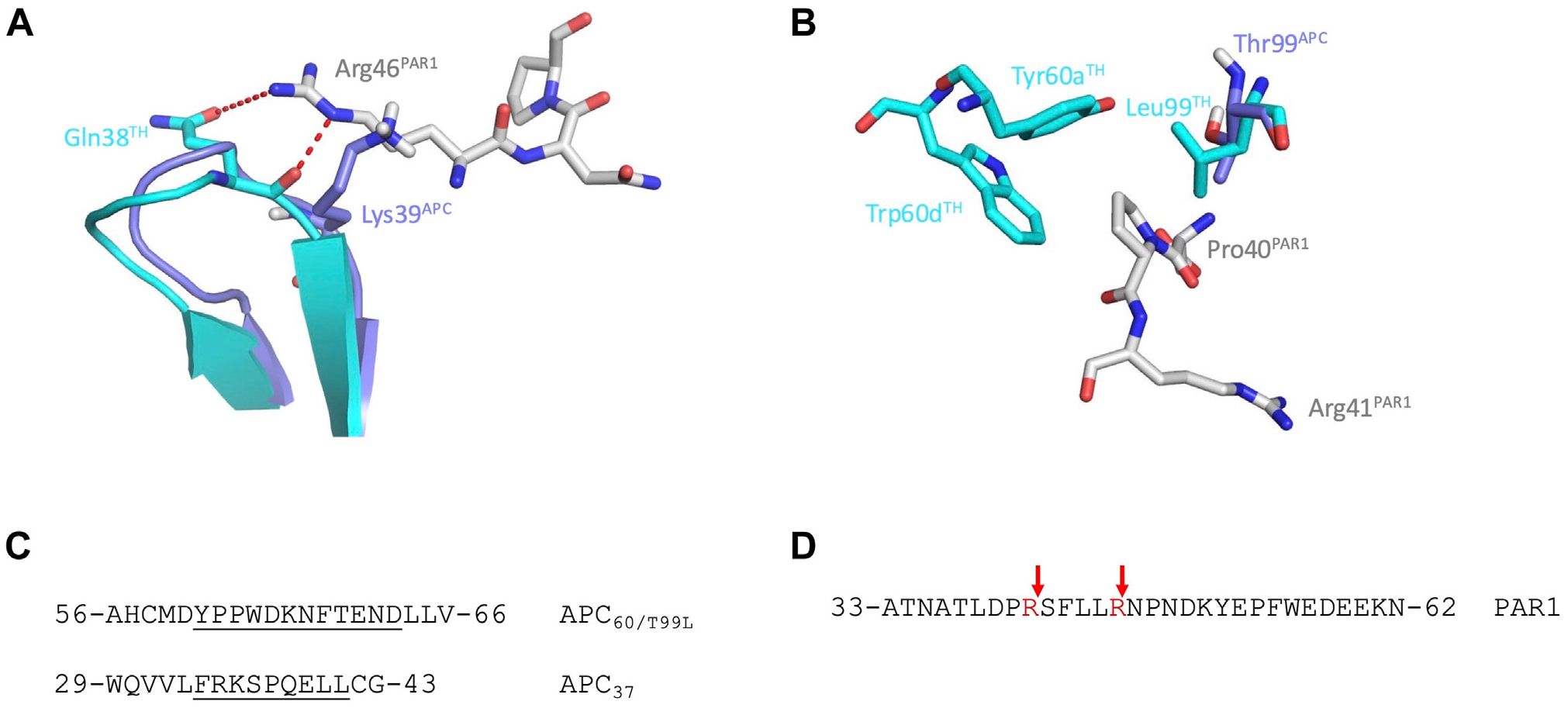
Overlay of the crystal structures of activated protein C (APC, purple; PDB 1AUT) and thrombin (cyan; PDB 3LU9) bound to the protease-activated receptor 1 (PAR1, gray). (A) The site of cleavage at R46 of PAR1 interacts with Q38 of thrombin but sterically clashes with K39 of APC. (B) At the S2 site of thrombin, the side chains of L99, Y60a, and W60d make hydrophobic interactions with P40 at the P2 position of PAR1. Similar interactions at the S2 site of APC are not favored by the polar side chain of T99 and shorter length of the 60-loop. (C) Amino acid sequence of the 37- and 60-loops of the engineered APC_37_ and APC_60/T99L_ mutants. APC_60/T99L_ was constructed by deleting the 215-ESKK-218 (mature PC numbering) sequence and inserting in its place the YPPWDKNFTEND sequence in a construct carrying the T99L mutation (chymotrypsin numbering). APC_37_ was constructed by deleting the 188-LDSKKKLA-195 (mature PC numbering) sequence and inserting in its place the FRKSPQELL sequence. APC_37/60/T99L_ carries the combined insertions from the APC_37_ and APC_60/T99L_ mutants. The replacements introduced by the swaps with thrombin are underlined. (D) Amino acid sequence of the fragment of PAR1 corresponding to the extracellular portion of the receptor. Cleavage sites at R41 and R46 are indicated by red arrows.

Thrombin cleaves PAR1 with values of *k*_cat_/*K*_M_ orders of magnitude faster than APC [6,7], and the difference likely depends on the determinants of PAR1 recognition in the 2 enzymes [10–15]. The structure of thrombin bound to the extracellular fragment of PAR1 reveals that R41 is positioned within the active site [11]. However, rather than binding to the active site, PAR1-R46 interacts with Q38 of thrombin, located in the 37-loop, which explains lack of cleavage at this site (Figure 1A). On the other hand, PAR1 cannot engage the 37-loop of APC in the same orientation as in thrombin due to steric clash between PAR1-R46 and K39 of APC (Figure 1A), which likely forces PAR1-R46 into the primary specificity pocket of APC for cleavage. Additional differences between the 2 enzymes involve the nature of the residue preceding the site of cleavage in PAR1, defined as the P2 residue [16], which is Pro (P40) for PAR1-R41 and Leu (L45) for PAR1-R46 (Figure 1D). Thrombin has a strong preference for Pro at P2 [17–19] because of the hydrophobic nature of the recognition site of this residue, referred to as the S2 site [16], which is defined by the side chain of L99 and a long insertion in the 60-loop containing Y60a and W60d (Figure 1B) [11,20,21]. These 2 residues are part of the 60-loop of thrombin, which contains a 12-residue insertion that constrains the size of substrate that can be accommodated in the S2 site. In contrast, the 60-loop of APC is by 8 residues shorter than in thrombin and poses no hindrance to the S2 subsite. Consequently, the analogous S2 site of APC is hydrophilic and solvent accessible because of the shorter 60-loop [22] and the presence of T99 that discriminates poorly between different residues at P2 [23] and would show low affinity for P40 in the R41 site of PAR1 (Figure 1B). The foregoing differences between thrombin and APC suggest a simple strategy to engineer PAR1 specificity in APC to be more like thrombin.

## 2 | METHODS

Expression and purification of PC were as described [24]. PC variants (8 μM) were converted into APC upon incubation with 0.3 μM thrombin for 90 to 120 minutes at 37 °C in 5 mM EDTA, 145 mM NaCl, 20 mM Tris, pH 7.5. After diluting NaCl < 50 mM, APC was loaded onto a Q-Sepharose (Cytiva) column equilibrated with 40 mM NaCl and 20 mM Tris, pH 7.5, and eluted using a 0 to 1 M NaCl gradient.

The active site concentration was quantified by monitoring the acyl-enzyme complex formation produced by reacting APC (100 nM) with the spectrofluorometric titrant 4-methylumbelliferyl 4-guanidinobenzoate (30 μM; Sigma–Aldrich) [25]. The release of 4-methylumbelliferone was continuously monitored (*λ*_excitation_ = 375 nm and *λ*_emission_ = 445 nm) under experimental conditions of 200 nM hirudin, 110 mM NaCl, 20 mM HEPES, pH 7.5, at 7 °C. The active site concentration was determined from the pre–steady-state burst formation of acyl-enzyme complex by referencing the change in fluorescence to a standard curve prepared with 4-methylumbelliferone (Sigma–Aldrich).

APC activity for the chromogenic substrates H-D-Val-Pro-Arg-p-nitroanilide (VPR) and H-D-Val-Leu-Arg-p-nitroanilide (VLR), each at 3.5 μM, was measured from progress curves of the release of p-nitroaniline at 405 nm. Values of *k*_cat_/*K*_M_ for cleavage of the soluble fragment of PAR1 (10 μM), corresponding to the extracellular portion of the receptor (Figure 1D), were measured by a high-performance liquid chromatography assay [10] using APC concentrations from 0.15 to 0.6 μM. All reactions were conducted in the presence of excess hirudin to eliminate possible cleavage by contaminating thrombin used to activate PC. Assays were performed under pseudo-first-order conditions with concentrations of PAR1 (10 μM) and VPR/VLR (3.5 μM) <*K*_M_ Experimental conditions were as follows: 200 nM hirudin, 145 mM NaCl, 5 mM CaCl_2_, 0.1% polyethylene glycol 8000, 20 mM Tris, pH 7.5, at 37 °C (PAR1) or 25 °C (VPR and VLR). Measurements were performed in triplicate.

Activated partial thromboplastin time (APTT) assays were used to determine the anticoagulant activities of the APC variants in plasma. Equal volumes (35 μL each) of normal plasma (King George Biomedical), STA-PTT A Reagent (Stago Diagnostica), and APC (10 nM) were preincubated at 37 °C for 5 minutes, and clotting was initiated by adding 35 μL of 25 mM CaCl_2_. Clotting times were measured on a Stago Start coagulometer, and each measurement was performed in triplicate.

Inactivation of human FVa (Haematologic Technologies) by APC was monitored by following the loss of cofactor function in an assay that measures the residual activity of prothrombinase. Variable concentrations of APC (0.16–10 nM) were added to a mixture of FVa (1 nM) and phospholipids (50 μM) in a final volume of 10 μL, and the reaction was incubated for 7 minutes at 20 °C. Each reaction sample was then mixed with 90 μL of a freshly prepared mixture composed of FXa (2 nM; Enzyme Research Laboratories), prothrombin (700 nM), and phospholipids (50 μM), followed by incubation for 3 minutes at 20 °C. The activation of prothrombin was quenched by adding 190 nM of apixaban, prepared in 10 mM EDTA/tris-buffered solution. After a 15-fold dilution of the reaction samples, the formation of thrombin was quantified by following cleavage of the chromogenic substrate H-D-Phe-Pro-Arg-p-nitroanilide at 405 nm. Experimental conditions were 20 mM Tris, 145 mM NaCl, 5 mM CaCl_2_, 0.1% bovine serum albumin, pH 7.5, at 20 °C. Each measurement was performed in triplicate.

## 3 | RESULTS AND DISCUSSION

**FIGURE 2:**
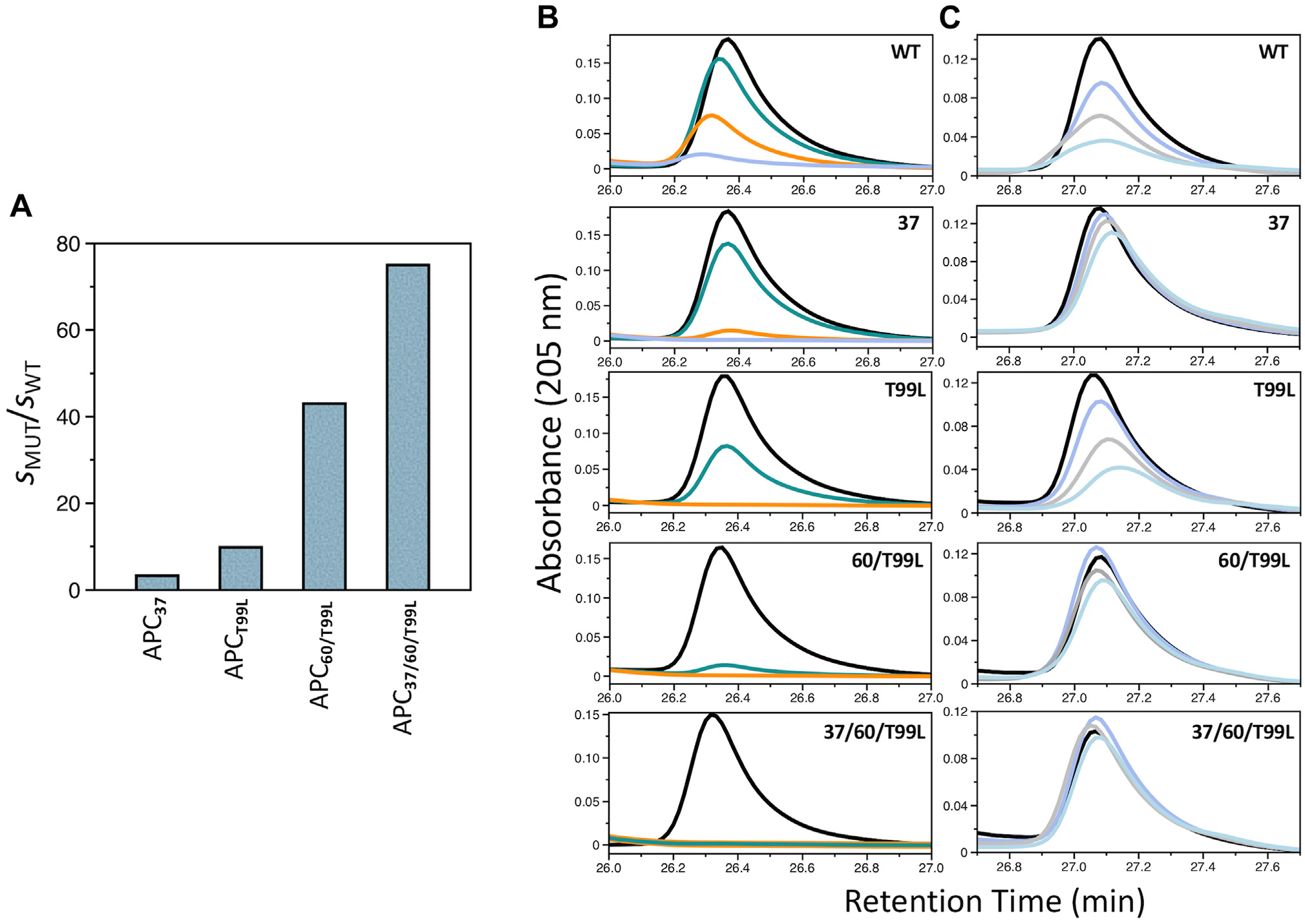
Cleavage of wild-type (WT) and R41A mutant of protease-activated receptor 1 (PAR1) by activated protein C (APC). (A) Values of s = *k*_cat_/*K*_M_ (Table) for the cleavage of the extracellular domain of PAR1. Ratios were calculated by dividing the rates of PAR1 cleavage by the specified APC mutant (MUT) over APC_WT_. Cleavage of (B) WT PAR1 and (C) PAR1_R41A_ (each peptide at 10 μM) by the specified APC variants (mutations are indicated in each panel) as monitored by a high-performance liquid chromatography assay. The concentration of all APC variants was 0.6 μM to facilitate comparison. Colors denote reactions quenched at 0 (black), 0.67 (teal), 8 (orange), 24 (purple), 48 (gray), and 72 hours (cyan). Experimental conditions were 200 nM hirudin, 145 mM NaCl, 5 mM CaCl_2_, 0.1% polyethylene 8000, 20 mM Tris, pH 7.5, at 37 °C. Residues that were mutated or inserted into specific APC mutants are defined in the legend of Figure 1.

Among the 2 possible sites of cleavage at R41 and R46 in the extracellular domain of PAR1, thrombin cleaves only at R41 with a rate that is nearly diffusion-limited, and APC cleaves at both sites with very low catalytic efficiency [6,7]. To better understand the molecular basis of this difference, several residues of APC were replaced with those of thrombin based on their specific interaction with PAR1 [11]. APC_T99L_ carries the T99L replacement in the S2 site, which is expected to introduce a hydrophobic interaction with P40 of PAR1. APC_37_ replaces the entire 37-loop of APC with that of thrombin to remove potential steric clash with PAR1-R46 (Figure 1C). The hydrophobicity and architecture of the S2 site of APC were further optimized for PAR1 recognition with the combined replacement of the entire 60-loop and the T99L mutation in the APC_60/T99L_ and APC_37/60/T99L_ variants (Figure 1C). Notably, the T99L mutation was found to be necessary for expression when swapping the 60-loop. The specificity of wild-type (WT) APC for the soluble fragment of PAR1 corresponding to the extracellular portion of the receptor is very poor and ~500 000-fold lower than that of thrombin [6,7,12]. Swapping the 37-loop produced a 3-fold increase in the value of *k*_cat_/*K*_M_, but introduction of the T99L mutation alone (APC_T99L_) or in combination with the 60-loop swap (APC_60/T99L_) produced increases of 10-fold and 40-fold, respectively (Table, Figure 2A). Addition of the 37-loop swap in the APC_37/60/T99L_ mutant increased *k*_cat_/*K*_m_ almost 80-fold (Table, Figure 2A).

Because thrombin only cleaves PAR1-R41, it is unclear how transferring structural elements of thrombin to APC may enhance specificity toward PAR1-R41 and compromise cleavage of PAR1-R46. To address this question, we tested the specificity of the mutants against a PAR1 peptide carrying the R41A mutation, PAR1_R41A_, which is cleavable only at PAR1-R46. Under conditions where cleavage of WT PAR1 by APC_37,_ APC_60/T99L_, and APC_37/60/T99L_ was completed, no significant cleavage of PAR1_R41A_ was observed up to 72 hours (Figure 2B, C). In contrast, both APC_WT_ and APC_T99L_ cleaved PAR1_R41A_ over this time frame. This suggests that the enhanced specificity toward PAR1 featured by the APC constructs, bearing the T99L mutation and swapping of the 37- and 60-loops with those of thrombin, comes from an increased preference for PAR1-R41 cleavage and compromised ability to cleave PAR1-R46. Swapping the S2 site of APC with that of thrombin in APC_60/T99L_ and APC_37/60/T99L_ compromises cleavage at PAR1-R46 and increases cleavage at PAR1-R41 up to 80-fold (Figure 2). Introducing the T99L mutation contributes to this effect, which is consistent with the contribution of residue 99 to the specificity of proteases involved in blood coagulation [23]. Swapping the 37-loop of APC with that of thrombin in APC_37_ compromises cleavage of PAR1 at PAR1-R46 and slightly enhances cleavage at PAR1-R41 (Figure 2). The result is likely a consequence of removing the steric clash predicted to occur between R46 of PAR1 and K39 of APC (Figure 1A) and introduction of favorable contacts with the 37-loop of thrombin [11].

The specificity of trypsin-like proteases is influenced by the nature of the P2 residue at the site of cleavage [26]. In the case of PAR1, this residue is Pro (P40) at the R41 site and Leu (L45) at the R46 site (Figure 1D). The Pro/Leu preference of the APC variants for the P2 position of substrate was further analyzed with the hydrolysis of the chromogenic substrates VPR and VLR (Table). APC_WT_ cleaved VPR and VLR with similar *k*_cat_/*K*_M_ values, but APC_T99L_ and APC_60/T99L_ showed nearly a 30-fold preference for VPR. Interestingly, APC_37_ was more effective than APC_WT_ in cleaving PAR1 but 4-fold less active toward VPR or VLR, suggesting that the 37-loop of thrombin introduced into the APC scaffold assumes a conformation better suited for PAR1 recognition. Similar effects were observed with the APC_37/60/T99L_ variant.

**FIGURE 3:**
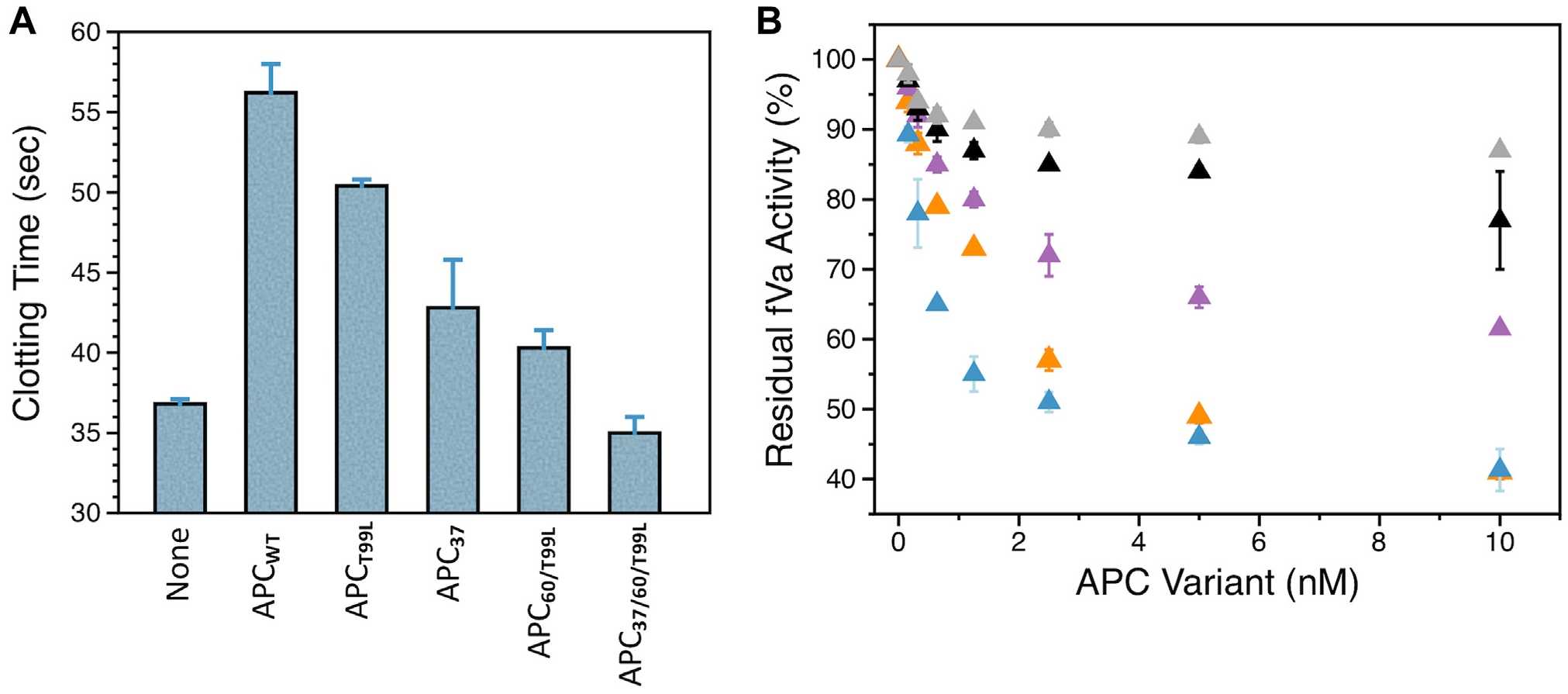
Anticoagulant activities of activated protein C (APC) variants. (A) Clotting times were measured by the activated partial thromboplastin time assay after incubation of normal plasma with 10 nM APC wild-type (WT) or mutants. (B) The extent of factor (F)Va inactivation by APC_WT_ (orange), APC_T99L_ (blue), APC_60/T99L_ (purple), APC_37_ (black), and APC_37/60/T99L_ (gray) was measured as residual cofactor activity in the prothrombinase assay. FVa (1 nM) was incubated with the indicated concentration of APC for 7 minutes under the following experimental conditions: 145 mM NaCl, 5 mM CaCl_2_, 0.1% bovine serum albumin, 20 mM Tris, pH 7.5, at 20 °C. Residues that were mutated or inserted into specific APC mutants are defined in the legend of Figure 1.

These mutations also compromise the ability of APC to inactivate FVa. The anticoagulant activity of APC in plasma was tested with the APTT assay, which measures the effectiveness of the clotting response. In agreement with previous reports [27], incubation of plasma with APC_WT_ significantly prolonged the clotting time due to increased inactivation of FVa and FVIIIa (Figure 3A). Prolongation of the clotting time measured in the presence of APC_T99L_ was slightly lower than APC_WT_, but that measured upon addition of APC_37,_ APC_60/T99L_, and APC_37/60/T99L_ was significantly compromised (Figure 3A). These findings confirm and expand on previous observations of compromised anticoagulant activity in APC variants carrying mutations in the 37- and 60-loops [27,28]. The results of the APTT assay are also consistent with independent measurements of APC cleavage of FVa, where APC_60/T99L_ was slightly less active than APC_WT_, while APC_37_ and APC_37/60/T99L_ were significantly compromised (Figure 3B).

All PC mutants were activated by thrombin at comparable rates in the absence of thrombomodulin, while in the presence of cofactor, the 37- and 60-insertions significantly compromised the *k*_cat_/*K*_M_ values (Supplementary Table). The results confirm the importance of these loops in promoting thrombomodulin binding reported previously [29].

We conclude that the increased hydrophilicity of the S2 site and unique residues of the 37-loop of APC are responsible for cleavage at PAR1-R46. Whether such APC mutants remain cytoprotective will await future cell-based and *in vivo* studies. We expect the cytoprotective signaling to depend on PAR1-R46 cleavage [7] and that the mutants will lack cytoprotective properties. However, if the cytoprotective signaling is initiated irrespective of the PAR1 cleavage site and depends only on binding of the Gla domain to EPCR [30], then the mutants might display cytoprotective features.

## Supplementary Material

1

The online version contains supplementary material available at https://doi.org/10.1016/j.jtha.2025.05.034

## Figures and Tables

**TABLE:** Specificity constants for cleavage of the protease-activated receptor 1, H-D-Val-Pro-Arg-p-nitroanilide, and H-D-Val-Leu-Arg-p-nitroanilide.

Enzyme	PAR1 *k*_cat_/*K*_m_ (mM^−1^s^−1^)	VPR *k*_cat_/*K*_m_ (mM^−1^s^−1^)	VLR *k*_cat_/*K*_m_ (mM^−1^s^−1^)
APC_WT_	0.061 ± 0.007	98 ± 6	40 ± 3
APC_T99L_	0.60 ± 0.03	200 ± 10	8 ± 1
APC_60/T99L_	2.6 ± 0.2	370 ± 40	13 ± 1
APC_37_	0.20 ± 0.005	26 ± 2	7.0 ± 0.2
APC_37/60/T99L_	4.6 ± 0.5	128 ± 8	3.0 ± 0.2

Experimental conditions were 200 nM hirudin, 145 mM NaCl, 5 mM CaCl_2_, 0.1% polyethylene glycol 8000, 20 mM Tris, pH 7.5, at 25 °C (VPR/VLR) or 37 °C (PAR1).

APC, activated protein C; PAR1, protease-activated receptor 1; VLR, H-D-Val-Leu-Arg-p-nitroanilide; VPR, H-D-Val-Pro-Arg-p-nitroanilide; WT, wild-type.
