# Supplementary material for: Structural determinants of protease-activated receptor 1 cleavage by activated protein C
Source: J Thromb Haemost. Author manuscript; Available in PMC 2026 May 25. (PMC13200511; doi:10.1016/j.jtha.2025.05.034)
Supplement: 1 [file NIHMS2177623-supplement-1.docx]

| Supplementary Table. Specificity constants for the activation of PC by thrombin | | |
| --- | --- | --- |
| Zymogen | **- Thrombomodulin**  *k*_cat_/*K*_m_ (mM^-1^s^-1^) | **+ Thrombomodulin***  *k*_cat_/*K*_m_ (mM^-1^s^-1^) |
| PC_WT_ | 0.18 ± 0.01 | 220 ± 20 |
| PC_T99L_ | 0.18 ± 0.02 | 125 ± 7 |
| PC_60/T99L_ | 0.11 ± 0.01 | 6.2 ± 0.6 |
| PC_37_ | 0.16 ± 0.02 | 6.3 ± 0.7 |
| PC_37/60/T99L_ | 0.25 ± 0.03 | 2.5 ± 0.2 |
| Experimental conditions: 145 mM NaCl, 5 mM CaCl_2_, 0.1% PEG8000, 20 mM Tris, pH 7.5 at 37°C  *200nM Thrombomodulin  Experiments were done at least in duplicates | | |
